# The French paediatric cohort of Castleman disease: a retrospective report of 23 patients

**DOI:** 10.1186/s13023-020-1345-5

**Published:** 2020-04-17

**Authors:** Charlotte Borocco, Claire Ballot-Schmit, Oanez Ackermann, Nathalie Aladjidi, Jeremie Delaleu, Vannina Giacobbi-Milet, Sarah Jannier, Eric Jeziorski, François Maurier, Yves Perel, Christophe Piguet, Eric Oksenhendler, Isabelle Koné-Paut, Caroline Galeotti

**Affiliations:** 1 Department of Paediatric Rheumatology, CeReMAIA, CHU Bicêtre, Assistance Publique - Hôpitaux de Paris, Université Paris-Sud-Saclay, 94270 Le Kremlin Bicêtre, France; 2 Department of Paediatrics, CHU Jean Minjoz, Besançon, France; 3 Department of Paediatric Hepatology, CHU Bicêtre, Assistance Publique -Hôpitaux de Paris, Le Kremlin-Bicêtre, France; 4 Paediatric Oncology Haematology Unit, Hôpital Pellegrin, Bordeaux, France; 5 Department of Internal Medicine, CeReMAIA, CHU Tenon, Assistance Publique - Hôpitaux de Paris, Paris, France; 6 Department of Paediatric Haematology and Oncology, Centre Hospitalier du Mans, Le Mans, France; 7 Department of Paediatric Haematology and Oncology, CHU Hautepierre, Strasbourg, France; 8 Department of Paediatrics, CeReMAIA, CHU Arnaud de Villeneuve, Montpellier, France; 9 Department of Internal Medicine, Hôpitaux privés de Metz, Metz, France; 10 Paediatric Oncology Haematology Unit, CHU de Limoges, Limoges, France; 11 Department of Clinical Immunology, CHU Saint-Louis, Paris, France; 12 National Reference Center for Castleman Disease, Paris, France

**Keywords:** Paediatric Castleman disease, Unicentric, Multicentric, Tocilizumab

## Abstract

**Background:**

Castleman disease (CD) is a rare non-malignant lymphoproliferation of undetermined origin. Two major disease phenotypes can be distinguished: unicentric CD (UCD) and multicentric CD (MCD). Diagnosis confirmation is based on histopathological findings in a lymph node. We attempted to survey all cases of paediatric CD identified to date in France to set up a national registry aiming to improve CD early recognition, treatment and follow-up, within the context of a new national reference center (http://www.castleman.fr).

**Methods:**

In 2016, we e-mailed a questionnaire to members of the French paediatric immunohaematology society, the paediatric rheumatology society and the Reference Centre for Castleman Disease to retrospectively collect cases of paediatric CD (first symptoms before age 18 years). Anatomopathological confirmation was mandatory.

**Results:**

We identified 23 patients (12 girls) with a diagnosis of UCD (*n* = 17) and MCD (*n* = 6) between 1994 and 2018. The mean age at first symptoms was 11.47 ± 4.23 years for UCD and 8.3 ± 3.4 years for MCD. The mean diagnosis delay was 8.16 ± 10.32 months for UCD and 5.16 ± 5.81 years for MCD. In UCD, the initial symptoms were isolated lymph nodes (*n* = 10) or lymph node associated with other symptoms (*n* = 7); fever was present in 3 patients. Five patients with MCD presented fever. No patients had HIV or human herpesvirus 8 infection. Autoinflammatory gene mutations were investigated in five patients. One patient with MCD carried a K695R heterozygous mutation in *MEFV*, another patient with MCD and Duchenne myopathy carried two variants in *TNFRSF1A* and one patient with UCD and fever episodes carried two heterozygous mutations, in *IL10RA* and *IL36RN*, respectively. Treatment of UCD was mainly surgical resection, steroids, and radiotherapy. Treatment of MCD included tocilizumab, rituximab, anakinra, steroids, chemotherapy, and splenectomy. Overall survival after a mean of 6.1 ± 6.4 years of follow-up, was 100% for both forms.

**Conclusion:**

Paediatric CD still seems underdiagnosed, with a significant diagnosis delay, especially for MCD, but new international criteria will help in the future. Unlike adult CD, which is strongly associated with HIV and human herpesvirus 8 infection, paediatric CD could be favored by primary activation of innate immunity and may affect life expectancy less.

## Introduction

Castleman disease (CD) or angio-follicular hyperplasia is a rare non-malignant lymphoproliferation of undetermined origin. CD diagnosis is difficult and often delayed because of insidious onset, low awareness and clinical heterogeneity. Two major disease phenotypes can be distinguished: unicentric CD (UCD), with involved lymph node(s) affecting a single station, and multicentric CD (MCD), with multiple lymph nodes and frequent inflammatory systemic symptoms [1]. The UCD phenotype is the most frequently reported in children and has the most favourable outcome [2].

Diagnosis confirmation is based on histopathological findings in an involved lymph node. The hyaline vascular (HV) type is characterized by abnormal germinal centres penetrated by hyalinised vessels and associated with abnormal follicular dendritic cells. The plasma cell variant (PCV) shows normal or enlarged germinal centres associated with interfollicular plasma cell infiltration. Symptomatic CD is often associated with increased production of interleukin 6 (IL-6) [3]. CD is also classified according to the presence or absence of human herpesvirus 8 (HHV-8); the association is clearly established in immune-compromised adults, the most frequent cause being HIV infection.

We attempted to survey all cases of paediatric CD identified to date in France to set up a national registry aiming to improve CD early recognition, treatment and follow-up, within the context of a new reference centre (http://www.castleman.fr).

## Methods

In 2016, we e-mailed a questionnaire to members of the French paediatric immunohaematology society, the French paediatric rheumatology society and the French Reference Centre for Castleman Disease to retrospectively collect information from medical charts of patients with paediatric CD (see Additional file 1).

We included all patients with a diagnosis of UCD or MCD who presented the first symptoms before age 18 years. Patients were required to have a diagnosis based on the pathological analysis of a lymph node biopsy of an affected area with the specific pathological criteria that we described previously.

Collected information included patient’s demographic information, age at first symptoms, age at diagnosis, clinical, biological and pathological findings, immunological profile with HHV-8 and HIV infection status, treatment strategy, clinical outcome and the speciality of the physicians involved. To better assess the burden and duration of the diagnostic delay, all other diagnostic procedures were reviewed (biopsies, CT scan, ultrasonography, PET scan, MRI, cytological puncture, myelography, endoscopy).

Genetic screening involved Sanger analysis for familial Mediterranean fever (*MEFV*) gene and/or by next-generation sequencing of a panel of 62 autoinflammatory disease genes (see Additional file 2).

According to French national regulations, no institutional review board approval was required for this retrospective study.

Cohort characteristics and other variables were analyzed with descriptive statistics (mean ± SD, number [%], range) and the Fisher exact test.

## Results

We identified 23 patients (12 girls) with a diagnosis of UCD (*n* = 17) and MCD (*n* = 6) between 1994 and 2018 (Table 1). The patients were seen in 14 centres and the diagnosis was established by paediatric haematologists (*n* = 9), adult immunologists (*n* = 6), paediatric rheumatologists (*n* = 6), a paediatric hepatologist (*n* = 1), an ENT specialist (*n* = 1), or a dermatologist (*n* = 1). Three cases were previously published [4–6].

**Table 1:** General clinical, laboratory and treatments characteristics of the paediatric cohort of unicentric CD (UCD) and multicentric CD (MCD)

	UCD (*n*=17)	MCD (*n*=6)
	mean±SD (range) or n (%)	mean±SD (range) or n (%)
Sex ratio (F:M)	9/8	3/3
Age at first symptoms (years)	11.47 ± 4.23 (0.25-16.5)	8.3 ± 3.4 (2.8-13)
Diagnosis delay (year)	0.68 ± 0.86 (0-3)	5.16 ± 5.81 (0-17)
Pathological type		
HV	13 (76.5%)	0 (0%)
PCV	3 (17.6%)	2 (33.3%)
Mixed type	0 (0%)	3 (50%)
No data	1 (5.9%)	1 (16.6%)
At diagnosis		
Fever	3 (17.6%)	5 (83.3%)
CRP level, mg/L	23.4 ± 42.07 (0.5-150)	50.68 ± 26.96 (7.1-96)
Hb level, g/dL	12.53 ± 2.52 (7.1-15.7)	10.23 ± 1.68 (8.8-13.6)
Platelet count, x10^9^/mm3	334.19 ± 151.34 (115-791)	319.17 ± 164.32 (141-665)
IgG level, g/L	12.8 ± 6.96 (6.9-29.7)	21.48 ± 7.69 (15-36)
HIV serology	0 (0%)	0 (0%)
HHV8 (serology, PCR or immunostaining)	0 (0%)	0 (0%)
Treatment		
Surgical excision	12 (70.6%)	0 (0%)
Radiotherapy	1 (5.9%)	0 (0%)
Chemotherapy	0 (0%)	1 (16.7%)
Tocilizumab	2 (11.8%)	5 (83.3%)
Anakinra	2 (11.8%)	1 (16.7%)
Steroïds	4 (23.5%)	3 (50%)
Splenectomy	0 (0%)	1 (16.7%)
No treatment	3 (17.6%)	0 (0%)
IgIV	1 (5.9%)	0 (0%)

*CD* Castleman disease, *F* female, *M* male, *HV* hyaline vascular, *PCV* plasma cell variant, *Hb* haemoglobin, *IgG* immunoglobulin G, *IgIV* intravenous immunoglobulin, *CRP* C-reactive protein, *HHV-8* human herpesvirus 8, *SD* standard deviation

**Fig. 1:**
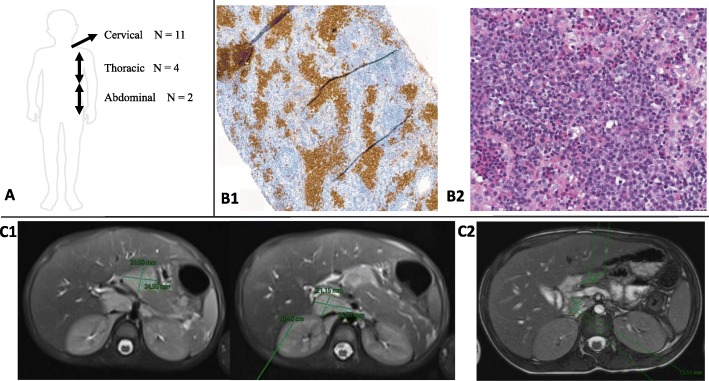
**A**: Adenopathy localizations in 17 patients with unicentric Castleman disease; **B**: Histopathologic findings in a multicentric CD patient with plasma cell variant, **B1**: CD138 immunohistochemical staining revealing interfollicular plasma cells, **B2**: hyperplastic interfollicular region of the node with sheets of plasma cells; **C**: Imaging findings in a 4-year-old patient with multicentric CD. **C1**: 2 MRI-detected intra-abdominal masses at diagnosis. **C2**: Decreased but persistent masses at 1 year of treatment with tocilizumab

For UCD patients, the mean age at first symptoms was 11.47 ± 4.23 years (range 0.25–16.5) and the mean diagnosis delay was 8.16 ± 10.32 months (range 0–36). The initial symptoms were isolated lymph nodes (10/17; 58.8%) or lymph node associated with other symptoms (7/17; 41.2%), and fever was present in only 3/17 (17.6%) patients (Table 2). Serum C-reactive protein (CRP) level was increased (> 10 mg/l) in 4/16 (25%) patients; the mean CRP level was 23.4 ± 42.07 mg/l (range 0.5–150). Elevated IgG level was observed in 4/12 (33%) patients; the mean IgG level was 12.8 ± 6.96 g/l (range 6.9–29.7). Mean haemoglobin level was 12.53 ± 2.52 g/dl (range 7.1–15.7) (16/17 patients) and mean platelet count 334.19 ± 151.34 × 10^9^/mm^3^ (range 115–791). Diagnostic investigations were lymph node biopsy (16/17; 94%), CT scan (13/17; 76.5%), ultrasonography (10/17; 58.8%), PET scan (7/17; 41.2%), lymph node cytological puncture (4/17; 23.5%), MRI (3/17; 17.6%), myelography (1/17; 5.9%), and upper and lower digestive endoscopy with digestive biopsies (1/17; 5.9%). The most frequent histologic finding on lymph node biopsy was the HV type (*n* = 13/17; 76.5%), then the mixed type (3/17; 17.6%). CD adenopathy was in the cervical area in 11/17 (64.7%) patients, intrathoracic in 4/17 (23.5%), and intraperitoneal in 2/17 (11.8%) (Fig. 1a).

**Table 2:** Clinical and laboratory features of 17 patients with UCD

Patient/Sex	Age at first symptoms (year)	Initial symptoms	Hb g/dl	platelets x10^9^/L	IgG g/l	CRP mg/l	ESR mm/h	Diagnosis delay (year)	Diagnostic investigations	Lymph node localization	Pathological type	Treatments	Follow-up time (year)	Evolution	Genetic variant
**P1 / M**	0.25	Cervical lymph node	11.9	371	8.12	-	-	0.9	Ultrasonography, CT scan, lymph node biopsy	Cervical	HV	Tocilizumab, steroids, anakinra, surgical excision	1.5	No relapse	ND
**P2 / F**	14	Right cervical lymph node	13	246	-	<4	10	0.5	CT scan, PET scan, lymph node biopsy	Cervical	HV	Surgical excision	1.5	No relapse	ND
**P3 / F**	10	Left cervical lymph node	12.8	304	-	6	4	2.5	Ultrasonography, CT scan, lymph node cytopunction, lymph node biopsy	Cervical	HV	Surgical excision	7	No relapse	ND
**P4 / M**	16.5	Oral pemphigus	14.9	248	14	3	-	0.5	CT scan, PET scan, lymph node biopsy	Thoracic	HV	Rituximab, steroids, immunoglobulin, surgical excision	3	No relapse	ND
**P5 / F**	14	Left cervical lymph node	12.8	310	-	0.6	-	0	Ultrasonography, CT scan, MRI, PET scan, lymph node cytopunction, lymph node biopsy	Cervical	HV	Steroids, Surgical excision	5	No relapse	ND
**P6 / F**	15	Torsion of an ovarian cyst and hepatosplenomegaly	7.1	791	21.2	89	95	0	PET scan, lymph node cytopunction	Thoracic	ND	Steroids, anakinra, tocilizumab and radiotherapy	8	Reduction of pulmonary lymph node after radiotherapy, which remains persistent, no relapse	ND
**P7 / M**	15.5	Fever and aseptic meningitis	-	-	-	49	61	0	CT scan, lymph node biopsy	Thoracic	MP	Surgical excision	11	No relapse but unexplained fever episodes, pericarditis	IL10RA: V406L/WT; IL36RN: S113L/W; MEFV: WT/WT
**P8 / M**	11.5	Fever and sporadic rectal bleeding	7.9	365	21	150	105	0.5	CT scan, lymph node biopsy	Peritoneal	MP	Surgical excision	8	No relapse	ND
**P9 / F**	6	Bronchopathy, 2 unilateral cervical lymph nodes	13.7	285	8.4	1	-	0	Ultrasonography, CT scan, lymph node biopsy	Cervical	HV	Surgical excision	0.75	No relapse	ND
**P10 / M**	13	Thoracic lymph node	14.3	237	9.2	2	-	0	CT scan, lymph node biopsy	Thoracic	HV	No treatment	18	Lymph node stability	ND
**P11 / F**	9	Cervical lymph node	13.8	115	6.9	1	-	1	Ultrasonography, CT scan, lymph node biopsy	Cervical	HV	Steroids	16	No relapse	ND
**P12 / F**	12	3 unilateral cervical lymph nodes	13.5	230	8.6	1	-	0	Ultrasonography, CT scan, lymph node biopsy	Cervical	HV	No treatment	2.8	Lymph nodes stability, coeliac disease	ND
**P13 / M**	15	Cervical lymph node	14.7	310	9.4	1	-	0	Ultrasonography, lymph node biopsy	Cervical	HV	Surgical excision	2.9	No relapse	ND
**P14 / M**	5	Fever, cervical lymph node evolving since 2 years	13.5	358	8.7	1	-	3	Ultrasonography, PET scan, lymph node biopsy	Cervical	HV	No treatment	0.5	Fever remission, lymph nodes stability	ND
**P15 / F**	10.3	Cervical lymph node	13	434	8.4	1	-	0.8	MRI, PET-Scan, lymph node biopsy	Cervical	HV	Surgical excision	0.7	No relapse	ND
**P16 / M**	15	Cervical lymph node	15.7	207	-	0.5	2	1.2	Ultrasonography, CT scan, lymph node cytopunction, lymph node biopsy, PET scan	Cervical	HV	Surgical excision	1	No relapse	ND
**P17 / F**	13	Abdominal pain, fatigue	7.9	536	29.7	65	61	0.7	Ultrasonography, CT scan, MRI, digestive endoscopy with biopsies, myelogram, lymph node biopsies	Peritoneal	MP	Surgical excision	3	No relapse	ND

*Hb* haemoglobin, *CRP* C-reactive protein, *ESR* erythrocyte sedimentation rate, *HV* hyaline vascular, *MP* mixed pathology, *ND* no data, *NGS* next-generation sequencing, *WT* wild type

Twelve of 17 patients underwent surgical lymph node excision (70.6%), 5/17 patients received steroids (29.4%), 3/17 (17.6%) patients received immunomodulatory treatments (tocilizumab = 2, anakinra = 2, rituximab = 1 and intravenous immunoglobulin = 1), 1/17 (5.9%) patient (P6) received radiotherapy and 3/17 (17.6%) patients had no treatments. At last evaluation after a mean follow-up of 5.33 ± 5.21 years (range 0.5–18), 12/17 patients were in complete remission (70.6%), 3/17 patients had a stable adenopathy size without treatment (17.6%), 1/17 (5.9%) patient had a persistent (but decreased) lesion after radiotherapy, and 1/17 (5.9%) patient (P7) still had recurrent fever after surgical resection of the adenopathy. P7 also experienced recurrent episodes of aseptic meningitis, pericarditis, neutropenia, lymphadenopathy, abdominal pain, persistent diarrhoea and interstitial lung disease. Screening for an autoinflammatory gene panel in this patient retrieved a class 2 (likely benign) heterozygous variant in *IL10RA* (V406L) and a pathogenic heterozygous variant in *IL36RN* (S113L) [7].

For patients with MCD (Table 3), the mean age at the first symptoms was 8.3 ± 3.4 years (range 2.8–13). They presented fever (5/6; 83.3%), abdominal lymph nodes (5/6; 83.3%), failure to thrive (3/6; 50%), hepatomegaly and/or splenomegaly (3/6; 50%), arthralgia (2/6; 33.3%), abdominal pain (2/6; 33.3%), fatigue (2/6; 33.3%), facial oedema (1/6;16.7%), isolated lymphadenopathy (1/6; 16.7%), rash on the trunk (1/6; 16.7%), vascular hepatopathy with oesophageal varicose veins (1/6; 16.7%), diarrhoea (1/6; 16.7%) and cholestasis (1/6; 16.7%). One patient (P23) had autism and Duchenne muscular dystrophy. Serum CRP level was increased in 5/6 (83.3%) patients; the mean CRP level was 50.68 ± 26.96 mg/l (range 7.1–96). Elevated IgG level was detected in 5/5 (100%) patients; the mean IgG level was 21.48 ± 7.69 g/l (range 15–36). The mean haemoglobin level was 10.23 ± 1.68 g/dl (range 8.8–13.6) and mean platelet count 319.17 ± 164.32 × 10^9^/mm^3^ (range 141–665).

**Table 3:** Clinical and laboratory features of 6 patients with MCD

Patient/Sex	P18 / F	P19 / F	P20 / M	P21 / F	P22 / M	P23 / M
**Age at first symptoms (years)**	13	7	6	11	10	2.8
**Initial symptoms**	Left jugular lymph node	Recurrent fever, arthralgia, hepatomegaly, splenomegaly, abdominal lymph nodes, failure to thrive, fatigue and facial edema	Fever, arthralgia, abdominal pain, abdominal lymph nodes and failure to thrive	Fever, abdominal lymph nodes	Recurrent fevers, hepatomegaly, splenomegaly, abdominal pain, abdominal lymph nodes, trunk rash, vascular hepatopathy and oesophageal varicose veins	Recurrent fevers, hepatomegaly, abdominal lymph nodes, failure to thrive, fatigue, diarrhea, cholestasis and Duchenne muscular dystrophy
**Haemoglobin level, g/dl**	13.6	9	9	8.8	10.1	10.9
**Platelet count, x10**^**9**^**/L**	261	328	270	250	141	665
**IgG level, g/l**	16	15	18	22.4	-	36
**CRP level, mg/l**	7.1	67	40	96	46	48
**ESR, mm**	20	55	75	-	-	131
**Leukocyte count, x10**^**9**^**/L**	7.4	10	-	7	8.1	14.9
**Initial diagnosis**	-	Primary parvovirus infection	Still disease then familial Mediterranean fever	-	unclassified vasculitis	-
**Initial treatment**	-	Colchicine	Aspirin, methotrexate, colchicine, corticosteroids	-	Corticosteroids, hydroxychloroquine, colchicine, NSAID, anakinra	-
**Diagnosis delay (years)**	0	3.5	7.5	1	17	2
**Diagnostic investigations**	PET scan, lymph node biopsy	Ultrasonography, CT scan, PET scan, liver biopsy, lymph node biopsy	CT scan, PET scan, lymph node biopsy	Ultrasonography, CT scan, lymph node biopsy	CT scan, PET scan, lymph node biopsy	Ultrasonography, CT scan, MRI, lymph node biopsy
**Histological type**	Mixed pathology	Plasma cell variant	Mixed pathology	Mixed pathology	ND	Plasma cell variant
**Treatments**	Tocilizumab	Tocilizumab	Chemotherapy (cyclophosphamide and vinblastine), rituximab, steroids, anakinra and tocilizumab	Steroids, splenectomy	Tocilizumab	Steroids, tocilizumab
**Follow-up time**	15 months	6 years	17 years	23 years	1 year	1 year
**Evolution**	Complete remission, no relapse at 3 months after the tocilizumab weaning	Patial remission, no relapse but persistence of hepatic hypermetabolic signals. Fluctuating lymphopenia and thrombocytopenia	Tocilizumab weaning after 4 years of treatment: increased inflammatory markers and headaches. Resumption of tocilizumab allowing for a disappearance of the symptoms. No relapse with tocilizumab	Complete remission, no relapse	Partial remission, no relapse	Partial remission, patient dependent on tocilizumab treatment. Appearance of non-specific inflammatory colitis.
**Genetic variant**	ND	*MEFV*: WT/WT	*MEFV*: K695R/WT	ND	*MEFV*: WT/WT	*TNFRSF1A*: P75L/P75L; *MEFV*: WT/WT

*CRP* C-reactive protein, *ESR* erythrocyte sedimentation rate, *NSAID* nonsteroidal anti-inflammatory drug, *CT* computerized tomography, *PET* positron emission tomography, *MRI* magnetic resonance imaging, *ND* no data, *NGS* next-generation sequencing, *WT* wild type

Diagnostic investigations were lymph node biopsy (6/6; 100%), CT scan (5/6; 83.3%), PET scan (4/6; 66.7%), ultrasonography (3/6; 50%), MRI (1/6; 16.7%) and liver biopsy (1/6; 16.7%). Other diagnoses considered before CD confirmation were primary parvovirus infection (1/6; 16.7%), familial Mediterranean fever (1/6; 16.7%), Still disease (1/6; 16.7%), and unclassified vasculitis (1/6; 16.7%). The histologic types of CD on lymph node biopsies were mixed subtype for 3/6 (50%) patients and PCV for 2/6 (33.3%) (Fig. 1b). The mean diagnostic delay was 5.16 ± 5.81 years (range 0–17). All 6 patients fulfilled the diagnosis criteria of idiopathic MCD proposed by Fajgenbaum et al. [8].

The *MEFV* gene was sequenced in 3/6 (50%) patients. P20 was heterozygous for K695R, and P19 and P22 had no mutations. All three patients showed no response to colchicine treatment. The genetic test in P23, with MCD, revealed a homozygous class 3 variant (P75L) in *TNFRSF1A*.

Patients received tocilizumab (5/6; 83.3%), steroids (3/6; 50%), chemotherapy (1/6; 16.7%), rituximab (1/6; 16.7%), anakinra (1/6; 16.7%) and splenectomy (1/6; 16.7%). The mean follow-up was 8.21 ± 8.69 years (range 1–23). Among the 5 patients who received tocilizumab, at last follow-up, P18 was in remission at 3 months after tocilizumab discontinuation, and 4 patients were still receiving tocilizumab: P19, P22 and P23 were in partial remission after 6 years, 1 year and 1 year, respectively, of tocilizumab, with decreased but persistent lymphadenopathies (Fig. 1c). P20 had a relapse upon discontinuation of tocilizumab after 4 years of treatment with inflammatory symptoms. Tocilizumab was then successfully reinitiated, with decreased but persistent lymphadenopathy. P21 was in remission after 1 year of steroids and splenectomy; she had no relapse after 23 years of follow-up.

All patients (23/23; 100%) were negative on HIV-1 and HIV-2 serology, and 22/22 (100%) were negative on HHV-8 serology, HHV-8 DNA PCR of blood or LANA1 staining (HHV-8 immunostaining) on biopsy.

## Discussion

Paediatric CD is an extremely rare disease, and its pathogenesis is poorly understood. We identified reference centres in France to gather one of the largest cohorts of paediatric CD reported so far, to build a national registry.

Our patients had an equal sex ratio and underwent much diagnostic wandering and delay. The 2 types of CD differed in delay, with mean diagnostic delay of 8.16 ± 10.32 (range 0–36 months) for UCD and 5.16 ± 5.81 years (range 0–17 years) for MCD. In comparison, in a reference centre for adult CD, the diagnostic delay was t 3 months for MCD and 5.6 months for UCD [9]. The main reasons for the diagnostic delay in CD are probably the lack of specificity of calling symptoms combined with little awareness of this condition among paediatricians as well as insufficient dialogue with pathologists. Unfortunately, the diagnostic delay has deleterious consequences such as increased morbidity due to chronic inflammation in children, particularly growth retardation, and significant burden related to useless explorations and untimely treatments.

Recently, a group of international experts published criteria for the diagnosis of idiopathic MCD that could help reduce the diagnostic delay [8]. These criteria include 2 major criteria: a standardized anatomopathological description and number of lymphadenopathies ≥2. Therefore, the diagnosis of MCD is based on a biopsy of a lesion and radiological staging on ultrasonography, CT scan, PET scan and MRI [10]. The minor criteria encompass many biological and clinical anomalies (11 criteria). Exclusion criteria are infection and oncologic and autoimmune diseases such as systemic lupus erythematosus. Despite these criteria, the differential diagnosis of autoimmune diseases is still difficult because autoantibodies (of systemic lupus erythematosus type) are found in about 30% of idiopathic CD cases [11]. All our cases of paediatric MCD satisfied these adult criteria of idiopathic MCD. In the future, fluorine-18-fluorodeoxyglucose-PET/MRI could have a role in staging, particularly in children because of the absence of irradiation with this technology as compared with PET or CT scan [12].

CD more likely presents as UCD (73.9–75% of cases) than MCD in children, whereas UCD represents 20.9% of CD cases in adult cohorts [2, 9]. Indeed, the disease mechanism may be different because most adult cases occur in a context of immunodeficiency associated with both HIV and HHV-8 infection, unlike in children. HHV-8–associated MCD represents a specific entity in terms of both treatment and prognosis. The association between CD and HHV8 has been reported only once in a child of consanguineous parents without HIV infection and living in an endemic country [13]. None of our patients was infected with HHV-8.

The role of IL-6 is important in the inflammatory manifestations of CD, which can mimic Still disease, another IL-6–related condition with underlying autoinflammatory mechanisms. IL-6 is secreted by the germinal centres of the lymph nodes in CD patients [3]. As a result, in our paediatric cohort, 83.3% of MCD patients had fever and mean CRP level of 50.68 ± 26.96 mg/dl (range 7.1–96).

Deregulation of the innate immune system may be critical in the pathogenesis of paediatric CD; this hypothesis was pursued by the investigation of autoinflammatory gene variants in five of our patients. Three underwent *MEFV* screening by Sanger analysis and two next-generation sequencing of a panel of autoinflammatory genes (additional file 2). Various sequence variants of unknown significance were retrieved in three different genes and in three of the five patients. In 2018, Van Nieuwenhove et al. reported a patient with MCD and adenosine deaminase 2 deficiency [14]. Overrepresentation of patients with autoinflammatory gene variants in paediatric MCD raises the possibility of amplified innate immune response to undefined triggers. Of note, systemic symptoms are also encountered in paediatric UCD and may be more frequent than in the adult counterpart: 17.6% with fever versus 4.2% without. However, this difference was not significant (*p* = 0.083). CRP level was also higher: 23.4 mg/dL (range 0–150) in children versus 2 mg/dL in adults [15]. Our results appeared to be similar to those observed in a recently published paediatric cohort of 24 patients [2] in which 44% of UCD patients had systemic symptoms.

The HV type is the most represented pathological type in paediatric UCD, 76.5%, as compared with adults, 68% [9]. Even if our patient cohort is small, paediatric patients may present more cervical lymph nodes than adults: 44 to 64.7% in children as compared with 26% in adults [2, 9].

In MCD patients, PCV and mixed types are the main pathological types in adults and children (77.7 and 83.3%) [9].

The treatment mainly depends on the CD type. Surgery is the gold standard for treatment of UCD and may be curative in 95% of cases [16]. A surgical approach may be compromised in certain sites of deep lymphadenopathy or located close to vessels. Pre-surgical treatment may be needed to facilitate total tumour excision, as in our patients P1 and P5, who received corticosteroids and biologic therapies (tocilizumab and anakinra) to reduce the size of cervical lesions before surgery. For unresectable locations, radiotherapy can be discussed, although its long-term toxicity remains an issue, particularly in children. A careful wait-and-watch strategy without treatment can also be proposed [15]. The approach to paediatric UCD in our cohort appeared to be the same as in adult CD, with 70.5% surgery (vs 66% in adults) and 17.5% wait-and-watch approach (vs 15% in adults). Only one child received radiotherapy (5.8%, vs 11% (8/71) in adults) [15].

Treatment of MCD, even if possibly not curative, is essential to limit serious complications of chronic inflammation and to improve quality of life [11]. Until recently, this treatment in adults as in children was not standardized. If surgery was not possible, steroids and chemotherapy were the first treatment used historically, but their efficacy was relatively limited, with a high cost of related toxicities. New therapeutic approaches have emerged, including anti-CD20, anti-IL-1 and anti-IL-6 biologic therapies [1, 5]. New guidelines were published in 2018 for adult idiopathic MCD, with siltuximab, an anti-IL-6 antibody, and tocilizumab, an anti-IL-6 receptor monoclonal antibody, now first-line treatments [17, 18]. For many years, tocilizumab was also used in paediatric CD [6]. In adult CD, tocilizumab allowed for reduction of lymph nodes to < 10 mm in only 52.2% of patients after 1 year of treatment [19]. In the same study, CRP and fibrinogen levels were normalized in 64.3 and 71.4% of patients, respectively, after 16 weeks of treatment. We also describe a suspension effect of tocilizumab on the disease in four children, with a complete response in terms of inflammatory symptoms. However, P20 showed a relapse of inflammatory symptoms after discontinuation of tocilizumab after treatment for 4 years, which then had to be resumed. For the other 3 patients, liver damage remained for P19, and decreased size lymphadenopathies persisted in P22 and P23. The question of weaning remains to be studied in children as in adults. However, one of our patients (P18) was in complete remission at 3 months after stopping therapy. Another study reported a total cure after combined chemotherapy followed by tocilizumab and discontinuation of all treatments [20]. The benefit of IL-6–targeting drugs in CD is not fully known because they have never been included in a comparative drug trial. Nevertheless, the therapeutic attitude in paediatrics now seems to be the same as in adults, with the use of tocilizumab as a first-line treatment for MCD.

Our patients showed 100% survival after a mean of 6 years of follow-up (range 1 month to 23 years), for all types of CD. In adults, the prognosis also seems good in UCD, close to 100%, but quite poor in MCD, with a 35% rate of death at 5 years [21].

## Conclusion

We report a large cohort of paediatric Castleman’s disease, in which, unlike in adults, the unicentric form was the most common. The new diagnostic criteria for idiopathic MCD should be tested in children to reduce the delay to diagnosis. The association of paediatric MCD with autoinflammatory gene variants, rather than HHV-8 and HIV infection, may not be incidental and suggests a primary deregulation of the innate immune system. Il-6–targeting drugs regularly eliminated inflammatory symptoms in our patients, but both treatment duration and long-term safety are still unknown.

## Supplementary information

**Additional file 1.** Paediatric Castleman disease data collection questionnaire

**Additional file 2.** Next-generation sequencing of a panel of 62 autoinflammatory disease genes.

## Data Availability

Please contact the corresponding author for data requests.

## Abbreviations

CD: Castleman’s disease
HHV-8: Human herpesvirus 8
HV: Hyaline vascular
MCD: Multicentric Castleman’s disease
PCV: Plasma cell variant
SD: Standard deviation
UCD: Unicentric Castleman’s disease
