# Supplementary material for: The French paediatric cohort of Castleman disease: a retrospective report of 23 patients
Source: Orphanet J Rare Dis. 2020 Apr 17;15:95. doi: 10.1186/s13023-020-1345-5 (PMC7164260; doi:10.1186/s13023-020-1345-5)
Supplement: Supplementary file 1 — Additional file 1. Paediatric Castleman disease data collection questionnaire [file 13023_2020_1345_MOESM1_ESM.pdf]

RETROSPECTIVE STUDY ON PAEDIATRIC CASTLEMAN DISEASE

1- Identity

Name (3 first letters) : ______________________ First name : (3 first letters) ______________________

Date of birth : ______________________ Gender : ______________________

First place of Follow-up

Hospital : ______________________ Department : ______________________

Referring physician : ______________________ Phone : ______________________

Mail : ______________________ Fax : ______________________

Specialization of the physician :

Paediatric haematologist ☐ Paediatric rheumatologist ☐

Rheumatologist ☐ Haematologist ☐

other ☐ :

Second place of Follow-up (if necessary)

Hospital : ______________________ Department : ______________________

Referring physician : ______________________ Phone : ______________________

Mail : ______________________ Fax : ______________________

Specialization of the physician :

Paediatric haematologist ☐ Paediatric rheumatologist ☐

Rheumatologist ☐ Haematologist ☐

other ☐ :

2– Family history :

Parental country of origin ______________________

Consanguinity ? Yes ☐ No☐ Unknown ☐ Degree :

3– Diagnosis

- Age at first symptoms ______________________

- Inital clinical symptoms :

☐ Lymph nodes

Number and localization: ______________________

☐ Fever, type

☐ Kidney involvement

☐ Association with systemic disease

☐ Lung injury, type :

☐ Skin involvement, type :

☐ Neurologic injury, type :

☐ Hepatic injury, type :

☐ Haematologic involvement, type :

☐ Cancer, type:

☐ Heart injury, type :

☐ Gut injury, type:

☐ Splenomegaly ☐ Hepatomegaly ☐ Failure to thrive

☐ Oedema / ascites ☐ Arthritis / arthralgia ☐ Asthenia

☐ Other :

- **Initial biological symptoms :**

· MEFV mutation: ☐ Yes ☐ No if yes type :

- Hb :
- Patelets :
- gammaglobulinemia :
- Fibrinogen :
- CRP :
- ESR :
- Albuminaemia :
- Natremia :
- Serum amyloid A protein:
- WBC count :
- Interleukin-6 rate:
- Proteinuria

- **Initial diagnosis (if different)** :
- **Treatments received before diagnosis (if different from those received after diagnosis):**
- **Age at Castleman disease diagnosis:**

- **Serology** :

HIV blood serology :	☐ positive	☐ negative	☐ not done or unknown
HHV8 blood serology :	☐ positive	☐ negative	☐ not done or unknown
HHV8 blood PCR assay :	☐ positive	☐ negative	☐ not done or unknown
HHV8 PCR assay on biopsy :	☐ positive	☐ negative	☐ not done or unknown

- **Additional examinations to diagnose Castleman disease** :

☐ Echography

☐ PET scanner

☐ Scintigraphy

☐ CT

☐ Biopsy, localization and Results:

___

___

___

___

- **Type of Castleman disease** :

☐ Unicentric

☐ Multicentric

☐ Hyaline vascular

☐ Plasma cell

☐ Mixed pathology

4- Treatments :

- **Treatment of first intention** :

☐ Surgical excision ☐ Radiotherapy

☐ Chemotherapy, type: ___

☐ Biotherapy, type : ___

☐ Corticosteroids ___

☐ Anakinra, ___

☐ Tocilizumab ___

☐ Other : ___

___

- Age at initiation of treatment: ___
- Duration : ___

- **Treatment of second intention** :

☐ Surgical excision ☐ Radiotherapy

☐ Chemotherapy, type: ____________________

☐ Biotherapy, type : ____________________

☐ Corticosteroids ____________________

☐ Anakinra, ____________________

☐ Tocilizumab ____________________

☐ Other : ____________________

____

- Age at initiation of treatment: ____________________
- Duration : ____________________

- **Treatment of third intention** :

☐ Surgical excision ☐ Radiotherapy

☐ Chemotherapy, type: ____________________

☐ Biotherapy, type: ____________________

☐ Corticosteroids ____________________

☐ Anakinra ____________________

☐ Tocilizumab ____________________

☐ Other ____________________

____

- Age at initiation of treatment: ____________________
- Duration : ____________________

5– Evolution

- Duration of follow-up **:**

- **Evolution** :
  - ☐ Remission, duration
  - ☐ Relapse then remission
  - ☐ Relapse(s)
  - ☐ Death
  - ☐ Complications: infections, macrophage activation syndrome, other :
