# Supplementary material for: The French paediatric cohort of Castleman disease: a retrospective report of 23 patients
Source: Orphanet J Rare Dis. 2020 Apr 17;15:95. doi: 10.1186/s13023-020-1345-5 (PMC7164260; doi:10.1186/s13023-020-1345-5)
Supplement: Supplementary file 2 — Additional file 2. Next-generation sequencing of a panel of 62 autoinflammatory disease genes. [file 13023_2020_1345_MOESM2_ESM.doc]

| *ACP5* | *ADAM17* | *ADAR* | *AP1S3* | *CARD14* | *CECR1* | *COPA* | *DDX58* | *FAM105B* | *FAS* |
| --- | --- | --- | --- | --- | --- | --- | --- | --- | --- |
| *FASLG* | *FBLIM* | *IFIH1* | *IL10* | *IL10RA* | *IL10RB* | *IL1RN* | *IL36RN* | *LACC1* | *LPIN2* |
| *MEFV* | *MVK* | *NCSTN* | *NLRC4* | *NLRP1* | *NLRP12* | *NLRP3* | *NOD2* | *PLCG2* | *POMP* |
| *PRF1* | *PSENEN* | *PSMA3* | *PSMB4* | *PSMB8* | *PSMB9* | *PSMG2* | *PSTPIP1* | *RBCK1* | *RNASEH2A* |
| *RNASEH2B* | *RNASEH2C* | *SAMHD1* | *SERPING1* | *SH3BP2* | *SLC29A3* | *TNEM173* | *TNFAIP3* | *TNFRSF11A* | *TNFRSF1A* |
| *TREX1* | *TRNT1* | *WDR1* | *XIAP* | *CASP1* | *RIPK1* | *LYN* | *MDFIC* | *RNF31* | *TNFRSF9* |
| *PYCARD* | *USP43* |  |  |  |  |  |  |  |  |
